# Prostate Tuberculosis Masquerading as Prostate Carcinoma: A Rare Case Report

**DOI:** 10.7759/cureus.30978

**Published:** 2022-11-01

**Authors:** Shalini Rawat, Anurag Singh, Akanksha Singh, Anuragani Verma, Mala Sagar

**Affiliations:** 1 Department of Pathology, King George's Medical University, Lucknow, IND; 2 Department of Microbiology, King George's Medical University, Lucknow, IND

**Keywords:** prostate carcinoma, antitubercular therapy (att), granulomatous inflammation, tuberculosis, prostate

## Abstract

Genitourinary tuberculosis (GUTB), the second most frequent type of extrapulmonary tuberculosis (TB) in endemic regions, was initially described by Wildbolz in 1973. The prostate and epididymis are the first sites of male genital tuberculosis, followed by the seminal vesicles and the testicles. Here, we describe a case of a 65-year-old male who presented with obstructive lower urinary tract symptoms (LUTS) for the previous six weeks. The digital rectal examination revealed prostatic enlargement with a firm and nodular surface. A high prostate-specific antigen level (88 ng/ml) was found in serum analysis. There was a suspicion of prostate cancer on the basis of clinical, radiological, and serological examination. In view of the suspicion of carcinoma, a prostate biopsy was performed, which revealed the proliferation of prostatic glandular and stromal elements with interspersed granulomas, necrosis, and aggregates of mature lymphoid cells. The histopathology findings were indicative of benign prostatic hyperplasia with granulomatous prostatitis. Ziehl-Neelsen (ZN) stain was negative for acid-fast bacilli. The cartridge-based nucleic acid amplification test (CBNAAT) for *Mycobacterium tuberculosis* was ordered on the prostate biopsy tissue bits, which showed positive results. On the basis of histopathology and nucleic acid amplification test, the diagnosis of prostate tuberculosis was considered. There are no specific clinical and radiological findings related to prostate tuberculosis; hence, the diagnosis can be established only after histopathological examination and tissue-based cartridge-based nucleic acid amplification test. Clinicians should have a high index of suspicion for tuberculosis, especially in patients from endemic countries who present with symptoms of the lower urinary tract, especially if there is granulomatous inflammation coupled with necrosis.

## Introduction

Genitourinary tuberculosis (GUTB), the second most frequent type of extrapulmonary tuberculosis (TB) in endemic regions, was initially described by Wildbolz in 1973 [1]. The prostate and epididymis are the first sites of male genital TB, followed by the seminal vesicles and the testicles. Since prostate involvement is relatively uncommon, prostate resection or biopsy specimens are typically examined histologically to make the diagnosis [2]. Here, we describe a rare case of benign prostatic hyperplasia with isolated prostate tuberculosis in a 65-year-old gentleman. There was a clinical suspicion of prostate cancer on the basis of clinical and radiological examination.

## Case presentation

A 65-year-old male had obstructive lower urinary tract symptoms (LUTS) for the previous six weeks, which included an increased frequency of micturition, dysuria, nocturia, and weak stream (International Prostate Symptom Score {IPSS}: 18). The uroflow rate was 9 ml/second. Hematuria, fever, stomach pain, breathing difficulties, loss of weight, and night sweats were not previously reported. The patient had no prior history of pulmonary tuberculosis or urethral instrumentation. The patient had no known TB family history. Vitals were found stable after a clinical assessment.

On physical examination of the chest, normal breath sound was heard over the chest with equal air entry in both lungs. No crepitation was noted. The digital rectal examination (DRE) revealed prostatic enlargement with a firm and nodular surface. A high prostate-specific antigen (PSA) level (88 ng/ml) was found in serum analysis. A complete blood hemogram revealed a hemoglobin level of 12.2 g/dl, total leukocyte counts of 7.2 × 10^9^/L, and platelet count of 310 × 10^9^/L with a normocytic normochromic population of red blood cells on the general blood picture. The urine bacteriological culture was sterile. Serology was negative for viral markers. The post-void residual (PVR) volume was 160 cc, and the prostate ultrasonography revealed a heterogeneous, enlarged 80 g prostate. There was a suspicion of prostate cancer on the basis of clinical (prostatic enlargement with a firm and nodular surface), radiological (heterochronic lesions in the peripheral zone), and serological examination (PSA level = 88 ng/ml).

**Figure 1:**
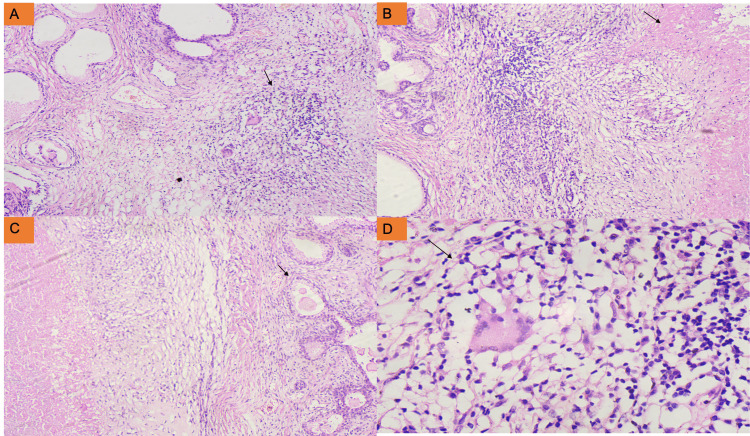
Histopathological examination of the prostate biopsy tissue (H&E stain) (A) Photomicrograph showing the proliferation of both the glandular and stromal elements of the prostate with interspersed granuloma (H&E stain: ×10). (B) Low-power view displaying the aggregates of mature lymphoid cells with necrotic areas (H&E stain: ×10). (C) Photomicrograph displaying the necrosis and glandular elements lined by the secretory and myoepithelial cell layer (H&E stain: ×20). (D) High-power view showing granuloma (H&E stain: ×40) H&E: hematoxylin and eosin

In view of the suspicion of carcinoma, an ultrasound-guided prostate biopsy was performed. The histological examination results revealed the proliferation of prostate glandular and stromal elements with interspersed well-formed granulomas, necrosis, and aggregates of mature lymphoid cells. These granulomas were composed of epithelioid histiocytes, Langhans-type multinucleated giant cells, and lymphocytes (Figure 1A-1D). The histopathology findings were indicative of benign prostatic hyperplasia with granulomatous prostatitis.

Ziehl-Neelsen (ZN) stain was negative for acid-fast bacilli in tissue biopsy. The cartridge-based nucleic acid amplification test (CBNAAT) for *Mycobacterium tuberculosis* was ordered on the prostate biopsy tissue, which showed positive results and sensitivity for rifampicin. On the basis of histopathology and nucleic acid amplification test, the diagnosis of prostate tuberculosis was considered.

The patient underwent a chest X-ray and a contrast-enhanced computerized tomography (CECT) scan of the thorax to rule out pulmonary tuberculosis, neither of which revealed any evidence of tuberculosis or interstitial lung disease. The sputum was negative for acid-fast bacilli and nucleic acid amplification test for *Mycobacterium tuberculosis*. The patient was started on antitubercular treatment for six months along with 0.4 mg/day tamsulosin for symptomatic management. Rifampicin, isoniazid, pyrazinamide, and ethambutol were given for two months and then two antibiotics, isoniazid and rifampicin, for four months. The follow-up was unremarkable after six months, with an improvement in LUTS (IPSS: 4), a significant reduction in PSA level (0.78 ng/ml), and a marked reduction in post-void residual volume (25 ml).

## Discussion

Genitourinary tuberculosis makes up 10%-14% of all extrapulmonary tuberculosis cases [3]. Isolated prostate tuberculosis is a very unusual and extremely rare extrapulmonary tuberculosis manifestation. The first case of prostate TB was described in 1882 by Jasmin [4]. It can be difficult to diagnose GUTB since its clinical signs and symptoms, such as LUTS and hematuria, can be mistaken for those of other conditions such as benign prostatic hyperplasia, prostate cancer, and urinary tract infections. Most prostate TB diagnoses are incidental and established by the pathologist while examining prostate biopsy; hence, careful search for granuloma is advised if there is the presence of foci of necrosis and aggregates of lymphocytes [5,6]. In cases of granulomatous prostatitis, if there are no acid-fast bacilli seen on the Ziehl-Neelsen stain, then the detection of *Mycobacterium tuberculosis* by CBNAAT is advised. The sensitivity and specificity of CBNAAT for the detection of *Mycobacterium tuberculosis* are 84.43% and 94.93%, respectively [7]. Hematogenous transmission of tuberculosis to the lower urinary system and kidneys is the most common route. Patient symptoms most commonly include frequent urination, nocturia, dysuria, hematuria, and urgency [8].

There were no unique clinical and radiological findings related to prostate tuberculosis; hence, the definitive diagnosis can only be established only after histopathological examination. Van Hau et al. reported a case of prostate tuberculosis in a 62-year-old male who was admitted to the hospital with acute urine retention and a three-month history of dysuria [9]. The prostate can show indurations and nodules on DRE, which makes it challenging to distinguish from malignancy. A case of tuberculous prostatitis that mimicked metastatic cancer was reported by Aziz et al. in an 80-year-old male who had sporadic low back ache for six months and a lower urinary tract obstruction [10]. The patient in our case had dysuria and LUTS symptoms.

The typical course of treatment consists of two months of intensive phase during which the patient takes four drugs (isoniazid 300 mg, rifampicin 600 mg, pyrazinamide 1200 mg, and ethambutol 2000 mg) on a daily basis, followed by a four-month to six-month continuation phase during which just two medications (isoniazid 300 mg and rifampicin 600 mg) are used, in a total of six to eight months. Extrapulmonary TB may typically be eradicated with appropriate therapy using conventional antitubercular drugs for six months, provided the TB is drug-susceptible [11]. In our case, a six-month TB drug treatment regimen was prescribed, and the symptoms were monitored every three months.

## Conclusions

Tuberculosis of the prostate is a rare disease that may mimic prostate carcinoma. For the definitive diagnosis of prostate tuberculosis, careful histological examination along with the application of Ziehl-Neelsen stain and nucleic acid amplification test should be performed. Clinicians should have a high index of suspicion for tuberculosis, especially in patients from endemic areas who present with symptoms of the lower urinary tract, especially if there is granulomatous inflammation coupled with necrosis and aggregates of lymphocytes.
